# An analysis of policy and funding priorities of global actors regarding noncommunicable disease in low- and middle-income countries

**DOI:** 10.1186/s12992-021-00713-4

**Published:** 2021-06-29

**Authors:** Kanykey Jailobaeva, Jennifer Falconer, Giulia Loffreda, Stella Arakelyan, Sophie Witter, Alastair Ager

**Affiliations:** Institute for Global Health and Development, Queen Margaret University, Edinburgh, EH21 6UU UK

**Keywords:** Global actors, noncommunicable diseases, funding, policies, and low and middle-income countries

## Abstract

**Background:**

Noncommunicable diseases (NCDs), including mental health, have become a major concern in low- and middle-income countries. Despite increased attention to them over the past decade, progress toward addressing NCDs has been slow. A lack of bold policy commitments has been suggested as one of the contributors to limited progress in NCD prevention and management. However, the policies of key global actors (bilateral, multilateral, and not-for-profit organisations) have been understudied.

**Methods:**

This study aimed to map the key global actors investing in action regarding NCDs and review their policies to examine the articulation of priorities regarding NCDs. Narrative synthesis of 70 documents and 31 policy papers was completed, and related to data collated from the Global Health Data Visualisation Tool.

**Results:**

In 2019 41% of development assistance for health committed to NCDs came from private philanthropies, while that for other global health priorities from this source was just 20%. Through a range of channels, bilateral donors were the other major source of NCD funding (contributing 41% of NCD funding). The UK and the US were the largest bilateral investors in NCDs, each contributing 8%. However, NCDs are still under-prioritised within bilateral portfolios – receiving just 0.48% of US funding and 1.66% of the UK. NGOs were the key channels of funding for NCDs, spending 48% of the funds from donors in 2019. The reviewed literature generally focused on NCD policies of WHO, with policies of multilateral and bilateral donors given limited attention. The analysis of policies indicated a limited prioritisation of NCDs in policy documents. NCDs are framed in the policies as a barrier to economic growth, poverty reduction, and health system sustainability. Bilateral donors prioritise prevention, while multilateral actors offer policy options for NCD prevention and care. Even where stated as a priority, however, funding allocations are not aligned.

**Conclusion:**

The growing threat of NCDs and their drivers are increasingly recognised. However, global actors’ policy priorities and funding allocations need to align better to address these NCD threats. Given the level of their investment and engagement, more research is needed into the role of private philanthropies and NGOs in this area.

**Supplementary Information:**

The online version contains supplementary material available at 10.1186/s12992-021-00713-4.

## Introduction

Noncommunicable diseases (NCDs) – namely cardiovascular diseases, cancer, chronic respiratory diseases, and diabetes – have become a major concern in low- and middle-income countries (LMICs) due to their rising prevalence contributing to premature mortality [1]. In 2016, 71% of 56.9 million global deaths were caused by NCDs. Three-quarters of NCD deaths (31.5 million) occurred in LMICs. Almost half of these deaths (46%) occurred before the age of 70 [2, 3]. Further, more than 80% of people experiencing mental disorders and substance abuse live in LMICs [4]. The increasing burden of NCDs, including mental health in LMICs, is putting strains on their struggling health systems as well as social and economic development [2, 5, 6].

In the past decade, NCDs have risen up the global agenda. High-level meetings of the UN General Assembly on NCDs were held in 2011, 2014, and 2018 [1]. As a result, important policy documents were developed: a Global Action Plan for the Prevention and Control of NCDs for 2013–2020 and an NCD Global Monitoring Framework (2013) [2]. NCDs were also included in the Sustainable Development Goals (SDGs). Goal 3.4 states: “by 2030 reduce by one-third premature mortality from noncommunicable diseases through prevention and treatment and promote mental health and well-being” [3]. The share of development assistance for NCDs has been slowly growing in the last decade (e.g. from 1.2% in 2010 to 1.7% in 2016) [5, 7].

However, a recent review of the progress towards SDG 3.4 showed that it was inadequate in most countries [3]. This issue is particularly acute in low-income and fragile settings. Hence, the review recommended that the international community increase financing and lending for the prevention and management of NCDs through bilateral and multilateral channels and through multi-donor funds and other innovative financing mechanisms [3].

For the most part, donors have been reluctant to make significant investments in NCDs, including mental health because (a) NCDs are not considered to be an immediate risk to others, and responsibilities lie with the individual behaviour [7, 8], (b) there is a dearth of data on the cost-effectiveness of interventions to address NCDs [7, 9–12], and (c) communicable diseases have established, low-cost strategies for management within the health sector which are easier to deliver than complex behaviour change strategies and a whole of society approach for NCD prevention and control which are multisectoral [7, 11].

Recently, Shilton [13] suggested that a lack of commitment to bold policies was an important reason for weak investment in NCDs. This point is of particular relevance given that donors play an important role in political guidance that influences agenda-setting and interventions at the national level [14, 15]. Examining their policies and funding can shed light on their commitments to NCDs.

This study examined the involvement of global actors (bilateral, multilateral, and not-for-profit organisations) in investment regarding NCDs in LMICs since 2010 by mapping the key actors and reviewing their policy documents and relevant literature to understand their commitment to NCD prevention and management. In particular, the study addressed three research questions: (a) who are the key actors and institutions investing in NCDs, and through which channels do they provide funding? and (b) to what extent are priorities regarding NCDs clearly articulated in the policies of donors and (c) how far do stated policy priorities and funding match one another?

## Methods

The study deployed a comprehensive literature search since 2010, a review of current policy documents from key global actors, and an analysis of the funding database for 2019.

### Literature review

A systematic search to identify relevant literature was undertaken in November–December 2019. The Cochrane Library, PubMed, Web of Science, PsychInfo, Scopus, and CINAHL were searched for published studies. Searches included terms to capture noncommunicable diseases (e.g. ‘noncommunicable disease’, ‘chronic disease’ and specific disease terms), global actors (e.g. ‘bilateral’, ‘multilateral’, ‘donors’), funding and policy terms (e.g. ‘funding’, ‘development assistance’, ‘policy’), and low- and middle-income countries. The full search strategy can be found in Additional file 1.

Studies were screened based on the eligibility criteria outlined in Table 1. Studies were included if they were published since 2010. The study focused on the last decade because NCDs had received increased attention in this period resulting in a number of UN high-level meetings, adoption of such key policy documents as the WHO Global Action for NCD prevention and Control 2013–2020, and importantly inclusion of NCDs into the SDGs.

**Table 1:** Criteria for selecting studies

Domain	Criteria
Time restriction	Study published since 2010
Language restriction	Study published in English
Population	Bilateral, multilateral, and international development organisations, international non-governmental organisations, international non-for-profit organisations including philanthropy of businesses and corporations
Topical focus	NCD funding, policies, and priorities
Study type	Qualitative and quantitative studies

Screening was undertaken by three researchers. Due to time constraints, results were single screened, however, frequent discussions were undertaken between researchers regarding inclusion and exclusion to ensure a shared understanding and consistent application of inclusion criteria. Additionally, one researcher reviewed all included studies to ensure inclusion criteria had been met.

Data extraction was undertaken by two researchers. The extraction form was designed in Excel and included information on: general identifiers, such as authors and year; study descriptors, such as setting, study purpose, and study focus; methodology, such as study design, population, and data source; relevant findings around funding and policy priorities; recommendations made by authors; and limitations identified. The extraction sheet was designed a priori and modified as required following piloting. The data extraction template can be found in Additional file 1.

### Funding Database

The Financing Global Health Data Visualisation Tool by the Institute of Health Metric and Evaluation (University of Washington) was used to obtain data on NCD and non-NCD funding sources and channels for 2019 to identify key funders of NCD action and the main actors in channelling NCD funds. This tool was selected from a number of tools explored (see Additional file 1) on the basis of comprehensiveness.

### Policy review

Based on the preliminary analysis of Financing Global Health Data, five key groupings of global actors were selected for the analysis of their policy documents: 1) UN agencies (namely WHO, UNDP, UNICEF, UNFPA), 2) Development Banks (i.e., World Bank (WB), Asian Development Bank (ADB), Inter-American Development Bank (IADB), African Development Bank (AFDB)), 3) European Commission, 4) Bilateral development partners and 5) the Bill and Melinda Gates Foundation and other philanthropies and foundations. Policy documents included policies, strategies, and policy briefs in relation to NCDs and health in general. If no NCD or health-related policies were found, foreign or development aid documents were included in the analysis. Policy documents were searched on the websites of these actors as well as in the Google search engine. The search terms included ‘noncommunicable’, ‘noncommunicable’, ‘health’, ‘human capital’, ‘foreign aid’, ‘development aid’, ‘strategy’, ‘policy’, and ‘policy brief’.

Data extraction of policy documents was undertaken by one researcher, using an additional data extraction template. Information was extracted on: the name of the actor; the title of the policy document; references to NCDs; and other health priorities indicated. The policy extraction template is available in Additional file 1.

### Analysis

Analysis across the included studies and policies of five global actor groups identified key themes around funding and policies in relation to NCDs and brought them together in a narrative synthesis. Research questions served as a key framework for the analysis. Data to examine the first research question (Who are the key actors and institutions in NCDs and through which channels do they provide funding?) was drawn from the Financing Global Health Database and studies identified through the literature search. The second research question (To what extent are priorities regarding NCDs clearly articulated in the policies of donors?) was answered through an analysis of policy documents and studies identified through the literature review. In the case of the policy document review, the analysis was done qualitatively by interpreting the meaning of the relevant text [16]. The third question was answered by comparing results across the first and second questions.

## Results

In addition to data obtained from the Financing Global Health Data Visualisation Tool, the literature search identified 70 studies. The majority of these studies were primarily general or global papers (n = 45); 12 studies had a regional focus (including Latin America and the Caribbean, the Gulf Region, sub- Saharan Africa, North Africa, and the Middle East, the Eastern Mediterranean, and East and South Asia); and 13 studies focused on one or more specific countries. These 13 studies included 37 different countries, the most frequent being India (in 10 studies), South Africa (7 studies), Uganda (6 studies), and Nigeria (5 studies). The majority of papers discussed NCDs – both physical and mental – as one group; however, 15 papers focused mostly on mental health, and nine mostly on physical aspects of NCDs. The search of key actors’ policies identified 31 policy documents. The majority of actors referenced in relation to NCDs were multilateral donors or actors (referenced in 76% of papers), followed by philanthropies and foundations (in 33% of papers), and bilateral donors or actors (in 27% of papers). Table 2 shows the number of mentions of NCDs for the three most frequently referenced actors within each of these categories. Private for-profit actors and public-private partnerships were mentioned in only five papers.

**Table 2:** Most frequently referenced actors in the reviewed literature according to type

Category	Actor	Mentions
Multilateral	WHO and WHO regional bodies	45
	UN and UN agencies (including UNICEF, UNAIDS, UNHCR)	13
	World Bank	11
Bilateral	USA	12
	UK	6
	Germany	4
Philanthropies and foundations	Bill and Melinda Gates Foundation	7
	Bloomberg Philanthropies	5
	Wellcome Trust	2

Policy documents included policies, strategies, and policy briefs in relation to NCDs and health in general. On average, two policy documents from each institution (*n* = 16) within the five actor groupings were included. Policy related documents from philanthropies were lacking. Online information from the website of the Gates Foundation, as the key foundation investing in health, including NCDs, was retrieved for the analysis. A full list of the documents analysed can be found in Additional file 1.

### Mapping of key global actors investing in NCD action

**Fig. 1:**
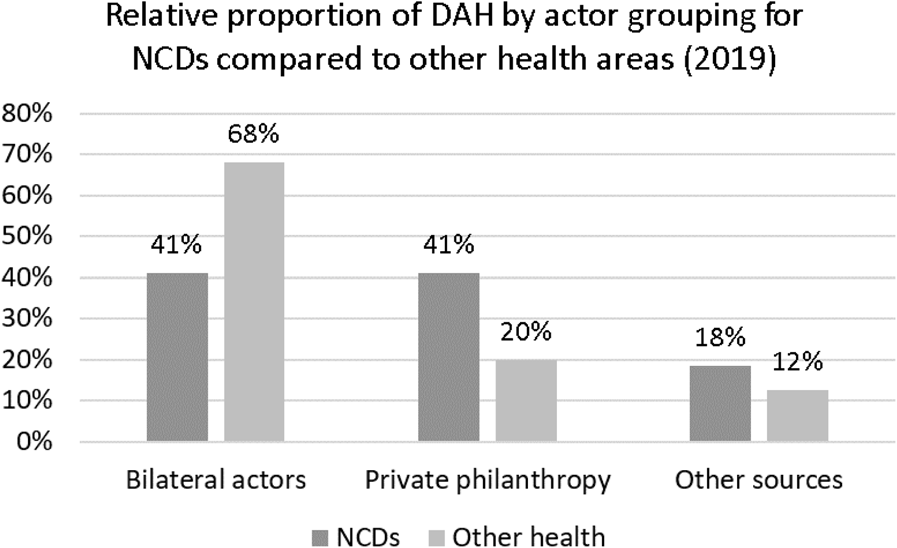
DAH in 2019 broken down by actor grouping for NCDs and other health areas (child and maternal health, HIV/AIDs, malaria, TB, and others)

**Fig. 2:**
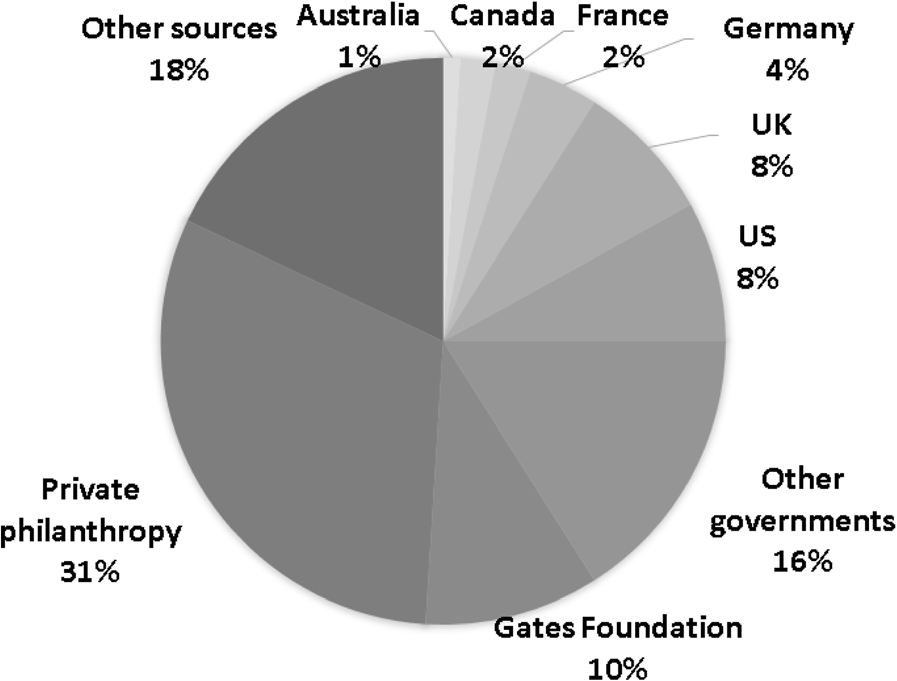
NCD funding sources in 2019

In 2019, a total figure of around $41 billion was spent on global health through mechanisms of Development Assistance for Health (DAH). About 1.81% of this ($ 733 million) was spent on NCDs. As Fig. 1 shows, 41% of DAH committed to NCDs came from private philanthropies, while philanthropic funding for other global health priorities was just 20%. Bilateral donors were the other major source of NCD funding (contributing 41% of DAH funds committed). This differs from the funding sources of other health areas (i.e., non-NCD) where most funding (68%) came from bilateral donors. Figure 2 shows that the UK and the US are the largest bilateral investors in NCDs, each contributing 8%. Other key bilateral investors include Germany, France, Canada, and Australia.

**Fig. 3:**
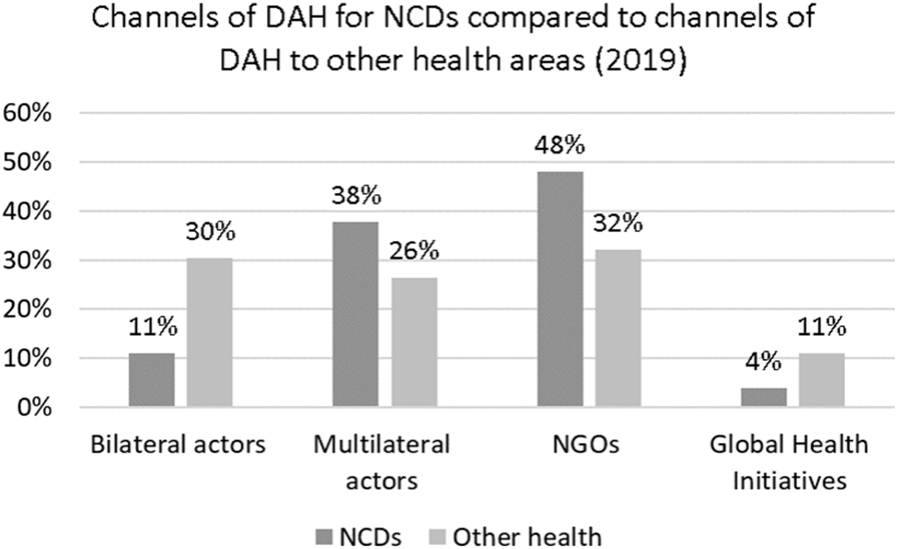
Channels of DAH for NCDs compared to channels of DAH to other health areas (child and maternal health, HIV/AIDs, malaria, TB, and others) in 2019. The category of NGOs also includes the Gates Foundation

**Fig. 4:**
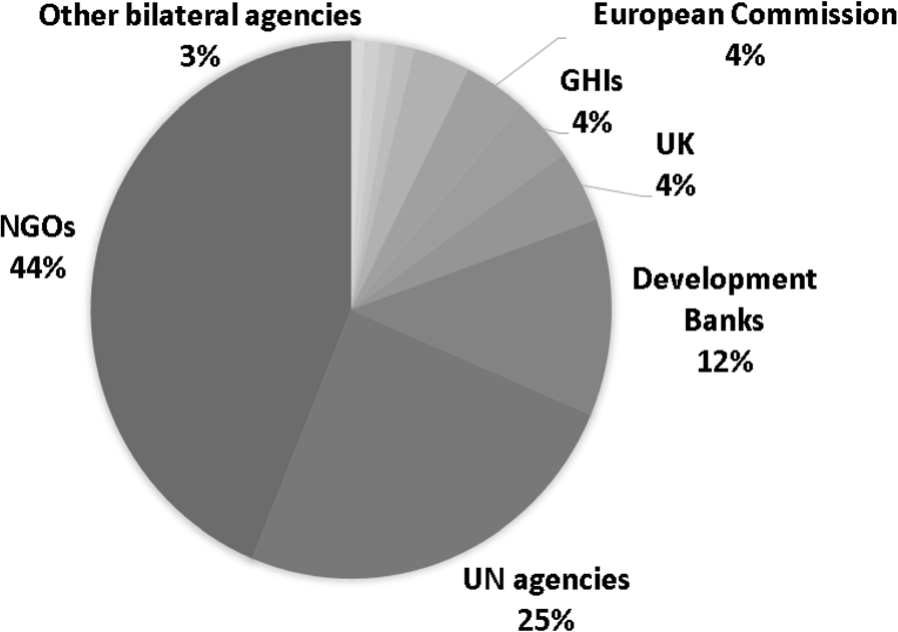
NCD funding channels in 2019

NCD funding is channelled through a range of organisations (see Figs. 3 and 4). NGOs are the key channels of funding for NCDs, utilising 48% of the funds from donors in 2019. A lower percentage of funds (32%) utilises NGO channels for non-NCD areas. Multilateral actors such as development banks and UN agencies are the second largest channel of funding for NCDs, also playing a proportionately larger role for NCD than other health expenditures.

**Fig. 5:**
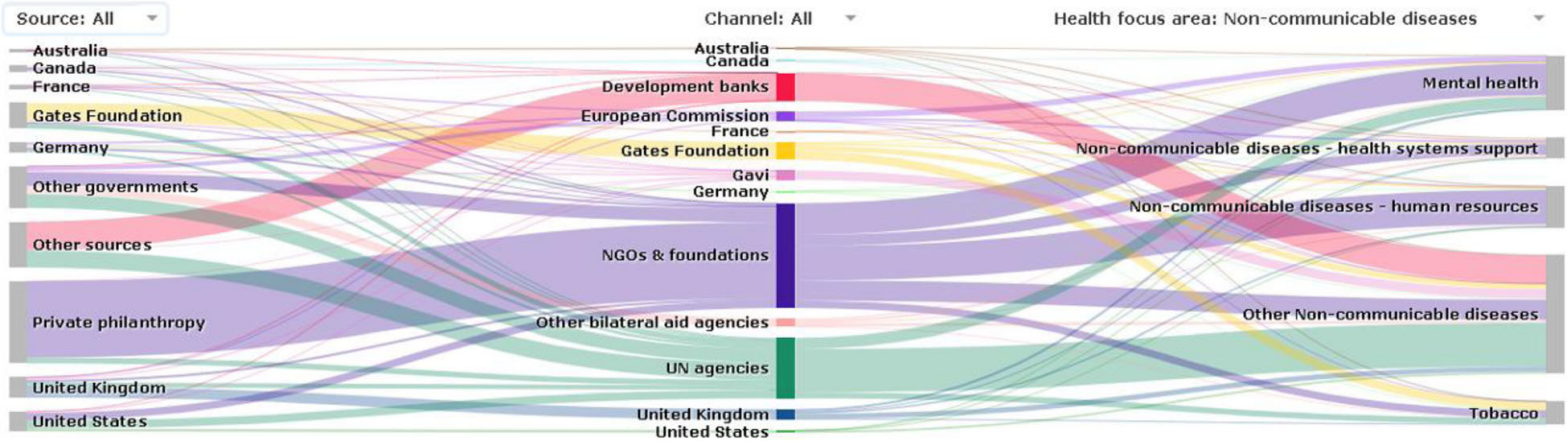
Sources and channels of NCD funding in 2019. Source: https://vizhub.healthdata.org/fgh/

NGOs, as a key channel for NCD interventions, receive funds from all sources, with private philanthropy and governments being the main funders. UN agencies, as the second important channel for NCD interventions, also receive funds from multiple sources, with bilateral and multilateral actors being the largest ones. Development banks, the third key channel, mostly disburse directly to governments (Fig. 5).

Private philanthropy and NGOs are important actors in NCD funding and action. Prominent philanthropies involved in NCD and mental health work identified in the reviewed literature include Bloomberg Foundation, GE Foundation [11, 14], MasterCard Foundation, Unilever Foundation, and Atlantic Philanthropies [8]. International NGOs engaged in NCD and mental health action include the World Heart Federation, the International Union against Cancer, Basic Needs, and Handicap International [12, 17–20].

### Review of policy documents

#### Published literature

The reviewed literature mostly discussed WHO policies related to NCDs in terms of the policies’ implementation and effectiveness. Key policies include the WHO Global Strategy for the Prevention and Control of Noncommunicable Diseases [19], WHO Global NCD Action Plan 2013–2020 [2, 21–26], WHO’s Framework Convention on Tobacco Control [1, 21, 27–31], WHO Mental Health Action Plan and Mental Health Gap [22, 32–34], WHO Strategy to Reduce Harmful Alcohol Consumption [26], WHO Best Buys [35], WHO Package of Essential NCDs Interventions for Primary Healthcare [35–37].

#### Bilateral actors

The US, UK, Germany, France, Canada, and Australia were identified as the main bilateral actors investing in NCDs, and our bilateral policy review was thus focused on documentation from these donors. Table 3 presents a) an overview of the NCD statements in the key policy documents identified for each funder, b) contribution of each donor to NCD funding in 2019, and c) NCD funding allocation within the health funding portfolio of each donor (i.e., internal allocation) in 2019.

**Table 3:** Overview of bilateral donors’ policy statements about NCDs and NCD funding contributions in 2019

Key bilateral donor	Key policy document	Funder policy statement about NCDs	NCD funding		Framing of NCDs
			Donor’s contribution to DAH on NCDs in 2019	Donor’s internal allocation to NCD action in health funding portfolio in 2019	
UK government	UK Aid: Tackling Global Challenges in the National Interest, 2015	Policy does not have any statements about NCDs	8% (58 million)	1.7% (58 million of 3.5 billion)	–
US government	National Security Strategy, 2017 USAID Policy Framework: Ending Need Foreign Assistance, 2019	Policy does not have any statements about NCDs	8% (57 million)	0.5% (57 million of 12 billion)	–
German government	Shaping Global Health Taking Joint Action Embracing Responsibility: The Federal Government’s Strategy Paper, 2014	Policy has a statement about NCDs	4% (29 million)	1.4% (29 million of 2.1 billion)	NCDs are identified as a global problem causing avoidable mortality and undermining opportunities for development, economic growth, social and political stability, and poverty reduction. Prevention and control of NCDs are explicitly stated under Policy Focus 3 “Expanding intersectoral cooperation – interaction with other policy areas.”
French government	France’s Strategy for Global Health, 2017	Policy has a statement about NCDs	2% (11 million)	1.5% (11 million of 760 million)	NCDs are recognised as a leading cause of mortality in the world and depleting health systems. NCDs are explicitly stated in Objective 1 under Priority 1. This objective aims to achieve UHC by promoting health systems that are accessible, durable, resilient, and of high quality using an integrated approach to communicable and noncommunicable diseases.
Canada government	Government website (Canada’s efforts to promote global health) www.international.gc.ca	Website does not have any statements about NCDs	2% (17 million)	1.6% (17 million of 350 million)	–
Australian government	Health for Development Strategy, 2015–2020	Policy has a statement about NCDs	1% (8.7 million)	2.5% (8.7 million of 350 million)	The increasing burden of NCDs is indicated as a factor weakening health systems that are already struggling to deal with infectious diseases and provision of quality maternal, newborn, and child healthcare. NCDs are stated under Priorities 1 and 4, which aim to invest in countries’ core public health systems and capacities and improved access to clean water, sanitation, hygiene, and nutrition to prevent and control NCDs.

As shown above, the UK contributed 8% to DAH on NCDs in 2019. Table 3 indicates that the UK is second in terms of NCD funding allocation within the health funding portfolio (1.7%). However, there is no mention of NCDs among health priorities in the UK Strategy for International Development as of 2015. Health priorities include infectious diseases including malaria, diseases of epidemical potential such as Ebola, neglected tropical diseases, drug-resistant infections, antimicrobial resistance, and disease outbreaks [38]. The strategy also aims to continue MDG commitments, namely child immunisation, nutrition, child and maternal death, and family planning. This strategy was developed on the outcomes of the 2015 Spending Review that assessed how the aid budget was spent. Only those projects that were identified to represent good value for money and in line with the UK objectives - such as reducing poverty and tackling global challenges that threaten British security and interests – are included [38]. In this exercise, prior commitment to addressing NCDs in the 2011–2015 policy appears to have been deemphasised [39].

The US also contributed 8% to DAH on NCDs in 2019. However, the US internally allocated the least (0.5%) to NCD action within its health funding portfolio among all actors reviewed (Table 3). The US’s current global health focus is primarily on child and maternal health, infectious diseases, and HIV/AIDS [40]. NCDs are not mentioned in the key policy documents that shape the foreign policy and international development agenda of the USA, namely the National Security Strategy (NSS; 2017, 42] and USAID policy framework [41]. In the NSS under the Trump administration, health has been mostly mentioned in terms of biothreats and pandemics (e.g., Ebola and SARS) [42]. The USAID policy framework indicates health to be important for people’s productivity to promote development and reach their country’s self-reliance, which is USAID’s new vision [41]. However, before Trump’s administration (January 2017 – January 2021), NCDs and global health in general were more salient in the US’s policy documents and political agenda [43]. Obama’s NSS referred to MDGs and SDGs and had a section on global health security (Obama’s administration dates – January 2009 – January 2017) [43]. Obama’s strategy laid the basis for the Global Health Initiative 2012–2016, which recognised NCDs as an emerging threat and health system strengthening as a way to tackle the NCDs [44]. Biden has called for strengthening the focus on health and healthcare [45]. On his first day of presidency, he halted a decision of the Trump administration to withdraw from WHO [46].

In 2019, Germany’s contribution to DAH on NCDs was 4%. The proportion of NCD funding within its health funding portfolio in 2019 was low (1.4%). However, unlike the UK and US, Germany has a global health strategy as of 2014, which identifies NCDs as a global problem causing avoidable mortality and undermining opportunities for development, economic growth, social and political stability, and poverty reduction in LMICs. The policy also recognises four factors, namely malnutrition, lack of exercise, tobacco consumption, and excessive alcohol consumption, as driving forces of NCDs’ rise. Thus, one of its focuses (Policy Focus 3) aims to promote a) prevention of NCDs with an emphasis on tobacco control, combatting harmful alcohol consumption, and promoting healthy eating and physical exercise, b) inter-sectoral cooperation, and c) strengthening health systems to prevent NCDs [47].

France contributed 2% to DAH on NCDs in 2019, which represents 1.5% of its health funding portfolio. Similar to Germany, France has a strategy for global health adopted in 2017, which recognises NCDs as a leading cause of mortality in the world and indicates that the growing burden of NCDs is depleting health systems [48]. The strategy of France, as the one by Germany, aims to take a preventive approach (tobacco, alcohol, nutrition, and physical activity), promote inter-sectoral cooperation, and strengthen health systems with a focus on testing, diagnostics, and management. Global strategies of both Germany and France emphasise the role of WHO in NCD action and express an intention to support NCD-related initiatives of WHO [47, 48]:

*“As part of the continuum of services, from prevention to care, France will implement the following actions: a) share its experience and help implement specific action plans (tobacco, alcohol, nutrition and physical activity) to encourage healthy lifestyles; b) step up tobacco control efforts, ensure that the WHO Framework Convention on Tobacco Control is applied, and fight addiction; c) encourage people to be more physically active, given that a sedentary lifestyle is a threat to global public health, and encourage targeted nutrition-focused actions; d) promote testing, diagnostics and the rapid management of diseases; e) advocate a graduated and inclusive service delivery ranging from local to specialist care; f) involve patients’ associations, economic stakeholders and healthcare workers in prevention and care.”* [48]

Despite contributing a similar percentage of DAH to NCDs as France (2%), which represents 1.6% of its health funding, Canada does not seem to have a policy on global health, including NCDs. The government website states that Canada’s global health priorities include diarrhoea, HIV/AIDs, Malaria, Polio, and TB, which are addressed through working with development partners to strengthen the health systems to ensure the provision of critical services, medicine, and interventions [49]. Since 2010, Canada has also focused on mental health through Grand Challenges Canada [50].

Australia’s contribution to DAH on NCDs in 2019 was the least (1%) among six donors. However, its internal allocation to NCD action was the highest (2.5%) among bilateral donors. The Australian Health for Development Strategy 2015–2020 recognises NCDs’ increasing burden, which weakens health systems that are already struggling to deal with infectious diseases and provision of quality maternal, newborn, and child healthcare. Thus, the strategy focuses on strengthening health systems and key partners’ capacity to strengthen chronic disease prevention, surveillance, and treatment system [51]. Geographically, the strategy focuses on Southeast Asia and the Pacific as Australia considers this region to be of direct relevance to Australian domestic interests [51].

### Multilateral actors

Table 4 provides a) an overview of the NCD statements in the key policy documents identified for each funder, b) contribution of each actor grouping (i.e., Banks and UN agencies) to NCD funding in 2019, and c) NCD funding allocation within health funding portfolio of each actor grouping (i.e., Banks and UN agencies) in 2019.

**Table 4:** Overview of multilateral donors’ policy statements about NCDs and NCD funding in 2019

Key multilateral donor	Key policy document	Funder policy statement about NCDs	NCD funding		Framing of NCDs
			Contribution to DAH on NCDs in 2019	Internal allocation to NCD action in health funding portfolio in 2019	
World Bank	Healthy Development: The WB strategy for Health, Nutrition, and Population Results (2007)	Policy has a statement about NCDs	11% (80 million)	3.1% (80 million of 2.6 billion)	NCDs are recognised as a major barrier to economic development and poverty reduction. The World Bank aims to invest in UHC and Health System strengthening to address NCDs.
Asian Development Bank	Strategy 2030: Achieving a Prosperous, Inclusive, Resilient, and Sustainable Asia and the Pacific	Policy has a statement about NCDs			NCDs are considered as a factor impeding economic development and poverty reduction. The ADB also aims to invest in UHC and Health System strengthening to address NCDs.
African Development Bank	At the Center of Africa’s Transformation Strategy for 2013–2022	Policy does not have any statements about NCDs			–
Inter-American Development Bank	Health and Nutrition Sector Framework Document: Social Protection and Health Division 2016	Policy has a statement about NCDs			NCDs are presented as an obstacle for economic development and poverty reduction. The Bank aims to tackle NCDs by raising awareness about healthy lifestyles, promoting access to high quality health services and nutrition, and promoting financial protection and efficient leadership and governance in the health section.
WHO	WHO Global Strategy for the Prevention and Control of Noncommunicable Diseases, Global NCD action Plan 2013–2020, Mental Health Action Plan 2013–2020	Policy has a statement about NCDs	6.7% (49 million)	3.5% (49 million of 1.4 billion)	WHO policy documents provide member states and international partners with a road map and menu of policy options to implement.
UNDP	UNDP HIV Health and Development Strategy 2016–2021	Policy has a statement about NCDs			NCDs are considered an impediment to development and poverty reduction. Priority 2.2 under Action Area 2 aims to strengthen governance to address NCDs and accelerate tobacco control.
UNICEF	UNICEF strategy for health 2016–2030 Programme Guidance for Early Life Intervention of NCDs	Policy has a statement about NCDs			NCDs are regarded as an impediment to children’s rights to health. NCDs are aimed to be integrated into policy actions on maternal, newborn, older child, and adolescent health, particularly in terms of nutrition and mental health. UNICEF also aims to strengthen health systems and promote integrated, multisectoral policies and programmes.
UNFPA	UNFPA strategic plan, 2018–2021	Policy does not have any statements about NCDs			–
EU	An introduction to the European Union’s International Cooperation and Development policy (2018)	Policy does not have any statements about NCDs	No data	No data	–

Funds for “Other Sources” in the health funding database were used in the table as funds invested by Banks and UN agencies. https://vizhub.healthdata.org/fgh/

11% ($ 80 million) of DAH on NCDs came from development banks, and only 3.1% (80 million of 2.6 billion of DAH from the Banks) was directed to NCD prevention and control activities (Table 4). Policies of most banks analysed have a reference to NCDs, which were recognised as a major barrier to economic development and poverty reduction. The World Bank and Asian Development Bank (ADB) invest in UHC and Health System strengthening to address NCDs [52–55]:

*“A significant increase has occurred in premature deaths related to chronic diseases (diabetes, pulmonary diseases, hypertension, cancer) linked to the tobacco-addiction and obesity pandemics. Malnutrition is problematic not only in poor countries (with both undernutrition and obesity), but also in rich countries confronted with a rapidly growing prevalence of obesity. … .. An increasing burden of NCDs in developing countries will put an enormous strain on their weak health systems. Sound health policies are essential, for example, to protect households from the impoverishing effects of catastrophic costs associated with NCD-related medical care. However, in countries with weak health systems, reaching the people needing these interventions through health service delivery mechanisms and improved basic nutrition is challenging. Strengthening health systems—so that these services can be delivered effectively, sustainably, and when needed—is critical to ensure that investments in health continue to foster economic growth to overcome poverty in generations to come”* [53]*.*

The Inter-American Development Bank (IADB) has a policy with the most elaborate focus on tackling NCDs by raising awareness about healthy lifestyles, promoting access to high-quality health services and nutrition, and promoting financial protection and efficient leadership and governance in the health sector [56]. The strategy of the African Development Bank (AFDB) does not have a reference to NCDs [57].

UN agencies contributed 6.7% of DAH on NCDs in 2019. Within their health funding portfolio, 3.5% were allocated for NCD prevention and control, which makes UN agencies a group with the greatest internal prioritisation of NCDs. WHO is the key actor at the international level, with a leadership and coordination role in promoting and monitoring global action against the rising burden of NCDs. The WHO Global Strategy for the Prevention and Control of Noncommunicable Diseases, Global NCD action Plan 2013–2020, and Mental Health Action Plan 2013–2020 provide member states and international partners with a road map and menu of policy options (i.e., best-buys) to implement various measures such as increasing tobacco taxes, reducing salt in food, and promoting access to affordable medicine:

*“The Global Action Plan provides Member States, international partners and WHO with a road map and menu of policy options which, when implemented collectively between 2013 and 2020, will contribute to progress on 9 global NCD targets to be attained in 2025, including a 25% relative reduction in premature mortality from NCDs by 2025. WHO and other UN Organizations will support national efforts with upstream policy advice and sophisticated technical assistance, ranging from helping governments to set national targets to implement even relatively simple steps which can make a huge difference, such as raising tobacco taxes, reducing the amount of salt in foods and improving access to inexpensive drugs to prevent heart attacks and strokes.”* [58]

The commitment of other UN agencies to NCD action in their policy documents varies. United Nations Development Programme (UNDP) appears to prioritise NCDs in its policies more than others, namely UNICEF and UNFPA, which appear to have recently expanded their focus to NCDs. As the UNICEF strategy states, after “the repeated calls for action, UNICEF has committed to integrating NCDs across programme sectors” [59]. UNDP discusses NCDs in its health policy as an impediment to development, and poverty reduction that deepens health-related disadvantages, exclusion, and inequality among different groups in the societies [60]. UNDP is supporting nations to develop their national NCD investment cases based on its partnership with WHO [61].

*“The social and economic burden of NCDs on the poor is rapidly growing. NCDs are now the single greatest cause of preventable illness, disability and mortality worldwide, and low- and middle-income countries bear a disproportionate burden. NCDs are by far the main cause of disability. For many people, intersecting vulnerabilities due to more than one ascribed or intrinsic identity, including gender, age, income, ethnicity, disability, sexual orientation and nationality, as well as indigenous, refugee, displaced or migratory status and religion or caste, may exacerbate health-related disadvantages, exclusion and inequality … UNDP partners closely with WHO to strengthen whole-of-government and whole-of-society NCD responses, including through implementation of relevant WHO-recommended approaches and agreements such as the WHO Global Action Plan for the Prevention and Control of Noncommunicable Diseases 2013–2020”* [60]*.*

UNICEF recognises NCDs in its policy as a barrier to children’s rights to health [59]. It is strengthening health systems and promoting integrated, multisectoral policies and programmes [59]. Even though UNFPA’s strategy does not make a clear reference to NCDs [62], UNFPA is committed to NCD work through promoting HPV vaccination, promoting cervical and breast cancer, healthy life choices, and strengthening mental health services to promote health of women and young people [63]. The EU’s International Cooperation and Development Policy makes no explicit reference to NCDs and identifies its health priorities as child and maternal health (i.e., nutrition, immunisation, reproductive health) and HIV/AIDS. Nonetheless, SDGs are presented as a backbone of the policy with an overall vision of the EU being to promote growth to end poverty and help partners achieve the SDGs, whilst being mindful of human rights and the availability of natural resources [64].

In the reviewed documents, multilateral actors emphasised that NCD action requires funding, which is very limited. There is competition for funding among multilateral actors and other actors. Value for money has been repeatedly mentioned as a key prerequisite for attracting funding. Further, multilateral actors stressed the need to increase public-private partnerships (PPP) with NGOs and other private actors to mobilise resources to tackle NCDs in the environment of economic challenges that affect flows of aid [52, 53, 59–61]. For example, WHO has developed a handbook for engagement with non-state actors, including NGOs [65].

#### Philanthropic foundations

Around 10% of NCD funding in 2019 was contributed by the Gates Foundation (highest among all actors reviewed) [66]. However, this represents only 1.84% of its health funding in 2019 [66]. No explicit policy document was found. The available information on the website of the Gates Foundation shows that it has four primary priorities: a) working across diseases to identify the public goods that accelerate global health impact and reduce the threat of epidemics; b) technical innovation to design, develop and deploy these public goods; c) vaccine development and manufacturing; and d) building high-quality modelling and forecasting capabilities informed by trustworthy primary data [67]. In relation to NCDs, the foundation supports HPV vaccination development and use [67]. The Gates Foundation, together with the Bloomberg Philanthropies and Canadian International Development Research Centre, have also started providing financial and technical support for tobacco prevention and control in Africa [29].

## Discussion

This paper analysed global actors’ funding and policy priorities regarding NCDs. To date, the literature has studied global actors’ NCD-related investment and policies separately. Concerning global actors’ NCD policies, the literature has predominantly focused on WHO policies related to NCDs, indicating a need to broaden the policy discussion [1, 2, 19, 22, 26, 29, 32–35, 37, 68–70]. Policies of other bilateral and multilateral actors have received little attention. Only a few available studies have assessed global health strategies of bilateral donors in general [50, 71]. With regard to funding, Nugent and colleagues have investigated NCD funding provided by bilateral, multilateral, and other international actors in the past decade by looking at funders, the chronology of aid, and funding volume [7, 14]. Recently, WHO analysed global spending on health, including NCD financing internationally and nationally [72]. This paper has taken a different approach by analysing global actors’ funding and policy priorities together.

By looking at funding and policy priorities conjointly, the analysis has identified a lack of alignment between policy and investment priorities of global actors. Priorities stated in the policies and funding allocations are weakly linked. Overall, there is a low prioritisation of NCDs despite a broad recognition that they are a growing threat. The current policies prioritise diseases that are infectious and of epidemic risk. NCDs do not seem to be seen to present the same global health security risks of communicable diseases and epidemics, which may be one of the factors in their deprioritisation. NCDs are predominantly seen by donors as a barrier to economic development, poverty reduction, and health system sustainability. Bilateral donors primarily focus on preventing NCDs with emphasis on curbing tobacco and excessive alcohol consumption and promoting nutrition and physical exercise. While these donors recognise the importance of multisectoral cooperation and health system strengthening in terms of surveillance, management, and care of NCDs, these are not fully spelled out in their policies.

Most multilateral actors articulate more elaborate actions in their policies, which are ultimately concerned with providing national governments with options of policies and interventions (e.g., best-buys and case investments) to prevent and control NCDs. However, uptake of these options by national governments has been limited, with prevention at the individual level being preferred, while costly interventions concerning NCD care are being overlooked [35, 73, 74]. This is mainly due to such factors as limited funding and capacity, and the challenge of tackling the interests of industries such as tobacco, alcohol, sugary beverages, and unhealthy food [23, 26, 28–30]. The applicability of the policy options offered by multilateral actors such as WHO best-buys to LMICs has also been questioned. The cost-effectiveness of best-buys from high-income countries cannot be fully transferred to LMICs as they do not have extensive health systems, while evidence from LMICs is lacking [25, 74–79]. Research capacity and infrastructure across LMICs remain inadequate, impeding the evidence generation needed for designing and implementing NCD interventions and policies in LMICs [35, 74, 80]. The distribution of investment regarding NCDs across actors and areas of health is inadequate to address NCDs’ threats in resources poor settings in particular. NCD support substantially comes from “non-traditional” actors, i.e., philanthropic foundations and NGOs, whose role and priorities are less transparent and less studied. The weak capacity of health systems in low-income countries (LICs,) particularly in fragile states, to deal with escalating rates of NCDs, the sequelae of that for their populations and development trajectories, and current patterns of DAH spending, especially the significant dependence on philanthropic funds, pose risks to a strategic response to NCDs.

Concerning NGOs, the analysis showed that NGO work on NCDs is under-researched. The limited literature indicates that NGOs engaged in NCD prevention and management are diverse, ranging from large alliances and associations to national not-for-profit organisations. In NCD tackling efforts, NGOs have contributed to policy development and interactions between various actors, technical support, capacity building, and resource mobilisation [8, 18, 24, 26, 27, 29, 81–83]. At the national level, NGOs, especially local ones, are involved in providing NCD services, including mental health, particularly to disadvantaged groups [22, 27, 84–87]. However, NGOs involved in NCDs and health systems struggle with sustainability as the local government structures still lack funding and the capacity to take over their activities [88]. Thus, their relevance to the local context is questioned as their approach appears to be top-down, driven by external agendas and expertise [88].

Drawing on the outcomes of this study, several avenues for future research can be stated. The first avenue is to expand policy analysis of prioritisation (or lack of it) within funders to understand the low allocations to NCDs, a major burden of illness group, and how those might be influenced. The second research direction is to investigate how NCD support is allocated internally, i.e., to what extent funding matches needs within the NCD arena and how this is influenced by funder priorities. The third avenue is to examine and understand how the international funder dynamics analysed by this study play out at the national level in terms of prioritisation of NCDs (overall and for specific components within them, including referral for diagnostics, curative and palliative care). Lastly, future research can explore agenda-setting in the philanthropic foundations and NGOs related to NCDs to understand their role and influence on NCD prevention and control in LMICs. A political economy lens would be appropriate to examine these three questions (Loffreda et al., forthcoming).

### Limitations

While our review constituted a comprehensive and rigorous search and screening of sources, a number of limitations should be highlighted. Firstly, as is common in policy document analysis, outdated policy documents had to be included. All effort was made to find the most up-to-date policy documents to mitigate this issue wherever possible. Additionally, while published studies were restricted to those published from 2010 onwards, studies from the literature search suffered the same limitation. All results were therefore viewed within their historical context. Secondly, studies in the analysis showed an anglophone bias due to the English language restriction in our eligibility criteria. Results must, therefore, be interpreted with this in mind. Thirdly, while our searches were comprehensive, there are likely documents, which were not available to us that have been overlooked. However, with 70 studies, 31 policy documents, and data from the Global Health Data Visualisation Tool included, the impact of any overlooked studies on our findings is likely minimal. Fourth, since no explicit category was available in the Financing Global Health Data Tool for multilateral actors among funding sources, we used data from two subcategories of “Other Sources” (Other-Development Banks and Other-UN Agencies) for analysing funding of multilateral actors covered in our study (i.e., World Bank, Asian Development Bank, African Development Bank, Inter-American Development Bank, WHO, UNDP, UNICEF, UNFPA). Further, we recognised the difficulties with disentangling NCDs from integrated programmes. To mitigate this, we used a specific category for NCDs available in the Global Health Data Tool. Finally, little information could be sourced on the names of philanthropies investing in NCDs – the Financing Global Health database did not provide the names of these, and information was also scarce in the published literature.

## Conclusion

This paper mapped the key global actors (bilateral, multilateral, and non-for-profit actors) investing in NCD prevention and management. It also reviewed their policies as a response to a recent statement that slow progress towards to SDG 3.4 may be due to a lack of commitment to bold policies. Key conclusions from the analysis include that DAH to NCDs remains limited, and there is a lack of alignment between stated policy goals and patterns of investment. There is also a low prioritisation of NCDs, notwithstanding a wide recognition of their expanding threat. The current allocation of NCD investment across actors is inadequate to address NCD challenges in resource-poor settings in particular. Lastly, philanthropic foundations and NGOs provide substantial support for NCD prevention and management, but their roles and priorities need further research.

## Supplementary Information

**Additional file 1.**

## Data Availability

The data that support the findings of this study are available from the corresponding author upon reasonable request.
