# Supplementary material for: An analysis of policy and funding priorities of global actors regarding noncommunicable disease in low- and middle-income countries
Source: Global Health. 2021 Jun 29;17:68. doi: 10.1186/s12992-021-00713-4 (PMC8240078; doi:10.1186/s12992-021-00713-4)
Supplement: Supplementary file 1 — Additional file 1. [file 12992_2021_713_MOESM1_ESM.docx]

# **Appendix A: Search Strategy**

| **Domain** | **Search Terms** |
| --- | --- |
| **NCD** | "Noncommunicable Disease" OR "Non-infectious Diseases" OR "Non infectious Diseases" OR "Non-infectious Disease" OR "Noncommunicable Diseases" OR "Disease, Noncommunicable" OR "Diseases, Noncommunicable" OR "Non communicable Diseases" OR "Noncommunicable Disease" OR "Noninfectious Diseases" OR "Noninfectious Disease" OR "Noncommunicable Chronic Diseases" OR "Chronic Disease, Noncommunicable" OR "Non communicable Chronic Diseases" OR "Noncommunicable Chronic Disease" OR "Chronic" OR "Cancer" OR "Tumor" OR "Neoplasm" OR "Cardiovascular" OR "Heart Disease" OR "Diabet*" OR "Chronic respiratory diseases" OR "Psych**" OR "Mental Health" |
|  | **AND** |
| **Global Actors** | "Bilateral" OR "multilateral" OR "non-for-profit" OR "donors" OR "development agency" OR "private organisation" OR "international NGO" OR "private foundation" OR "WHO" OR "Gates Foundation" OR "US government" OR "World Bank" OR "Pan American Health Organisation" OR "Inter-American Development Bank" OR "Welcome Trust UK" OR "Bloomberg Foundation" OR "GE foundation" OR "National institute of Health" OR "pharmaceutical companies" OR "pharmac*" OR "Russia" OR "China" OR "India" OR "Brazil" OR "Emerging donors" OR "private organisations" OR "USAID" OR "United States Agency for International Development" OR "DFID" OR "Department for International Development" OR "GIZ" OR "German Society for International Cooperation" OR "SIDA" OR "Swedish International Development Cooperation Agency" OR "AFD" OR "French Aid for Development" OR "Australia" or "Japan" |
|  | **AND** |
| **Concept of interest** | "funding" OR "assistance" OR "development assistance for health" OR "health technical assistance" OR "development assistance" OR "donor funding" OR "health finance" OR "program*" OR "intervention*" OR "promotion" OR "prevention" OR "control" OR "advocacy" OR "priority" OR "support" OR "implementation mechanism" OR "public-private partnership" OR "capacity building" OR "campaign" OR "strategy" OR "policy" |
|  | **AND** |
| Low- and Middle- Income Countries | “Afghanistan” OR “Albania” OR “Algeria” OR “American Samoa” OR “Angola” OR “Argentina” OR “Armenia” OR “Azerbaijan” OR “Bangladesh” OR “Belarus” OR “Belize” OR “Benin” OR “Bhutan” OR “Bolivia” OR “Bosnia and Herzegovina” OR “Botswana” OR “Brazil” OR “Bulgaria” OR “Burkina Faso” OR “Burundi” OR “Cabo Verde” OR “Cambodia” OR “Cameroon” OR “Central African Republic” OR “Chad” OR “China” OR “Colombia” OR “Comoros” OR “Congo” OR “Costa Rica” OR “Cote d'Ivoire” OR “Cuba” OR “Djibouti” OR “Dominica” OR “Dominican Republic” OR “Ecuador” OR “Egypt” OR “El Salvador” OR “Equatorial Guinea” OR “Eritrea” OR “Eswatini” OR “Ethiopia” OR “Fiji” OR “Gabon” OR “Gambia” OR “Georgia” OR “Ghana” OR “Grenada” OR “Guatemala” OR “Guinea” OR “Guinea-Bissau” OR “Guyana” OR “Haiti” OR “Honduras” OR “India” OR “Indonesia” OR “Iran” OR “Iraq” OR “Jamaica” OR “Jordan” OR “Kazakhstan” OR “Kenya” OR “Kiribati” OR “Democratic People’s Republic of Korea” OR “Kosovo” OR “Kyrgyz Republic” OR “Lao PDR” OR “Lebanon” OR “Lesotho” OR “Liberia” OR “Libya” OR “Madagascar” OR “Malawi” OR “Malaysia” OR “Maldives” OR “Mali” OR “Marshall Islands” OR “Mauritania” OR “Mauritius” OR “Mexico” OR “Federal state of Micronesia” OR “Moldova” OR “Mongolia” OR “Montenegro” OR “Morocco” OR “Mozambique” OR “Myanmar” OR “Namibia” OR “Nauru” OR “Nepal” OR “Nicaragua” OR “Niger” OR “Nigeria” OR “North Macedonia” OR “Pakistan” OR “Papua New Guinea” OR “Paraguay” OR “Peru” OR “Philippines” OR “Romania” OR “Russian Federation” OR “Rwanda” OR “Samoa” OR “Sao Tome and Principe” OR “Senegal” OR “Serbia” OR “Sierra Leone” OR “Solomon Islands” OR “Somalia” OR “South Africa” OR “South Sudan” OR “Sri Lanka” OR “St. Lucia” OR “St. Vincent and the Grenadines” OR “Sudan” OR “Suriname” OR “Syrian Arab Republic” OR “Tajikistan” OR “Tanzania” OR “Thailand” OR “Timor-Leste” OR “Togo” OR “Tonga” OR “Tunisia” OR “Turkey” OR “Turkmenistan” OR “Tuvalu” OR “Uganda” OR “Ukraine” OR “Uzbekistan” OR “Vanuatu” OR “Venezuela” OR “Vietnam” OR “West Bank and Gaza” OR “Yemen” OR “Zambia” OR “Zimbabwe” OR “East Asia and Pacific” OR “Eastern Europe” OR “Central Asia” OR “Latin America” OR “the Caribbean” OR “Middle East” OR “North Africa” OR “South Asia” OR “Sub-Saharan Africa” OR “Central America” OR “East Asia” OR “Eastern Asia” OR “East Africa*” OR “Eastern Africa*” OR “West Africa*” OR “West of Africa” OR “Western Africa*” |

# **Appendix B: Data Extraction Templates**

**Literature**

| **Domain** | **Extraction Item** | **Instruction** |
| --- | --- | --- |
| **Identifiers** | First author | Note surname, first initial of the first author |
|  | Other authors | Note surname, first initial of remaining authors separated by ; |
|  | Year | Note year of publication |
|  | Title | Note title of publication |
| **Primary descriptors** | Setting | Note the study focuses on  - global (LMIC)  - multi-region  - regional  - multi-country  - national |
|  | Region/Country | Note which regions and countries study focuses on. |
|  | Purpose of study | Paraphrase study purpose |
|  | Dates of Study | Dates over which study was carried out (if relevant) |
|  | Primary focus | Physical health  Mental health  Financing/funding NGOs/civil society  NCDs in general  NR |
|  | Secondary focus | Physical health  Mental health  Financing/funding NGOs/civil society  NCDs in general  NR |
| **Methodology** | Study design | Review  Commentary  Primary data collection  Secondary data analysis  Other (specify below) |
|  | Study type | 1. If 'other' selected above, please describe the study design 2. If primary data collection, please note whether the study was qualitative or quantitative or mixed |
|  | Population | If population groups are mentioned, please note which groups are mentioned |
|  | Data source | Please state key databases and references used in the analysis |
| **Relevant Findings** | Category of donor | Please choose which category donor in the discussion belongs to: Multilateral  Bilateral  Foundations and NGOs Other (please specify) |
|  | Name of donor | Please note the name/s of organisations/institutions |
|  | Funding facts | Note any facts and figures about NCD funding including mental health |
|  | Funding priority factors | Note:   1. Any factors that influence funding priorities 2. Any arguments criticising current funding streams and priorities |
|  | Policy facts | Note any policies on NCD including mental health |
|  | Policy priority factors | Note:   1. Any factors that influence policy priorities 2. Any points discussing policies (including criticism) |
|  | Civil society | Note any discussion, criticism, suggestions with regard to involvement of civil society, NGOs, CBOs, grass-root organisations, social movements, faith based organisations, faith actors (e.g. faith leaders,) informal self-help groups, womens' associations, youth associations, farmers' associations, trade associations' trade unions, informal actors (e.g. community leaders, traditional leaders, traditional healers). In short, any voluntary organisations and associations |
|  | Mismatched global/national priorities | Note any discussion of mismatch of global and national NCD priorities |
|  | NCD Needs -National | Note any discussion of national NCD needs |
|  | NCD - Infectious disease | Note any discussion about NCDs and infectious disease (particularly how these two can be integrated or what can be learned from interventions on infectious disease. |
| **Limitations and place in literature** | Limitations - author specified | Note any study limitations raised by authors |
|  | Limitations - reviewer note | Note any study limitations you believe applicable |
|  | Research gaps highlighted | If authors mention research gaps to be addressed, please paraphrase them |
| **Recommendations** | Recommendations | Please state any recommendations made in the article for future funding and policy priorities |
| Notes | Notes | Please state any additional notes if needed |

**Policy Documents**

| **Extraction Item** |
| --- |
| 1. Actor name |
| 1. Document title |
| 1. Document year |
| 1. Is NCD explicitly stated in the document (Yes/No) |
| 4.a If yes, what explanations are given for having NCDs as a priority? |
| 4.b What is the extent of NCD discussion in the policy? Insert extracts from the policy. |
| 4.c What other health priorities are indicated in the document? |
| 4.d What explanations are given for having these health priorities? |
| 1. Important quotes |

# **Appendix C: Funding Databases Explored**

| **Database name** | **Website** |
| --- | --- |
| PHR National Health Accounts | <http://www.phrplus.org/abnha.html> |
| Resource Flows Database | <http://resourceflowsdata.org> |
| AiDA (Accessible Information on Development Activities), | <http://rdi.developmentgateway.org/#/?_k=vagbum> |
| World Development Indicators | <https://datacatalog.worldbank.org/dataset/world-development-indicators> |
| Financing Global Health Data Visualisation  Institute of Health Metrics and Evaluation | <https://vizhub.healthdata.org/fgh/>  <http://www.healthdata.org/health-financing> |

# **Appendix D: Policy Documents Reviewed**

| **No** | **Actor** | **Document title** | **Document year** |
| --- | --- | --- | --- |
| 1 | UK government | 2010-2015 government policy: health in developing countries | 2010, updated in 2015 |
| 2 | UK government | UK aid: tackling global challenges in the national interest | 2015 |
| 3 | UK government | Department of Health and Social Care Annual Report and Accounts | 2018-2019 |
| 4 | UK government | Public Health England: Global Health Strategy | 2014 |
| 5 | US government | National Security Strategy | 2017 |
| 6 | US government | National Security Strategy | 2015 |
| 7 | German government | Shaping Global Health Taking Joint Action Embracing Responsibility: The Federal Government's Strategy Paper | 2014 |
| 8 | French government | France's strategy for global health | 2017 |
| 9 | Australian government | Health for Development Strategy | 2015-2020 |
| 10 | USAID | USAID's Global Health Strategic Framework: Better Health for Development | 2012-2016 |
| 11 | USAID | 50 years of Global Health Report | 2016 |
| 12 | USAID | USAID Policy Framework: Ending Need Foreign Assistance | 2019 |
| 13 | World Bank | Responding to the challenge of NCDs: the WB (Policy Brief) | 2019 |
| 14 | World Bank | Healthy Development: The WB strategy for Health, Nutrition, and Population Results (2007) | 2007 |
| 15 | UNDP | Responding to the challenge of NCDs: UNDP (Policy Brief) | 2019 |
| 16 | UNDP | UNDP HIV Health and Development Strategy 2016-2021 | 2016 |
| 17 | EU | An introduction to the European Union's International Cooperation and Development policy (2018) | 2018 |
| 18 | ADB | Strategy 2030: Achieving a Prosperous, Inclusive, Resilient, and Sustainable Asia and the Pacific | 2018 |
| 19 | ADB | ADB Health Bond: Continuing to meet regional demand for strategic health financing | 2019 |
| 20 | AFDB | The Bank's Human Capital Strategy for Africa 2014-2018 | 2014 |
| 21 | AFDB | At the Center of Africa's Transformation Strategy for 2013–2022 | 2013-2022 |
| 22 | AFDG | Harmonisation for Health in Africa | 2006 |
| 23 | IADB | Health and Nutrition Sector Framework Document: Social Protection and Health Division | 2016 |
| 24 | IADB | Institutional Strategy 2010-2020 | 2010 |
| 25 | WHO | Global Action Plan for the Prevention and Control of NCDs 2013-2020 | 2013 |
| 26 | WHO | Mental Health: Action Plan 2013-2020 | 2013 |
| 27 | UNFPA | UNFPA strategic plan, 2018-2021 | 2017 |
| 28 | UNFPA | Responding to the Challenge of Noncommunicable Diseases: UNFPA (Policy Brief) | 2019 |
| 29 | UNICEF | UNICEF strategy for health 2016 - 2030 | 2016 |
| 30 | UNICEF | Programme Guidance for Early Life Intervention of NCDs | 2019 |
| 31 | Gates | Online strategy overview | 2020 |
